# Ureteroenteric strictures: a single center experience comparing Bricker versus Wallace ureteroileal anastomosis in patients after urinary diversion for bladder cancer

**DOI:** 10.1186/s12894-019-0529-6

**Published:** 2019-10-24

**Authors:** Frank Christoph, Franziska Herrmann, Peter Werthemann, Thomas Janik, Martin Schostak, Christian Klopf, Steffen Weikert

**Affiliations:** 1 University of Urology, Otto-von-Guericke-Universität, Magdeburg, Leipziger Str. 44, 39120 Magdeburg, Germany; 2 Department of Urology, Vivantes Humboldt Klinikum Berlin, Am Nordgraben 2, 13509 Berlin, Germany

**Keywords:** Bricker, Wallace, Ureteroenteric anastomosis, Stricture, Hydronephrosis

## Abstract

**Background:**

To evaluate the outcome and complication rate in a single institution experience using the two most commonly used techniques of ureteroenteric anastomosis, the Bricker and Wallace anastomosis.

**Methods:**

A total of 137 patients underwent ileal conduit for bladder cancer. Ureters were anastomosed by two experienced surgeons, one performing a Bricker and the other, a Wallace anastomosis. Stricture was identified during clinical follow-up.

**Results:**

Seventy-five patients underwent a Bricker anastomotic, and 65 received a Wallace anastomosis. The average age was 70 in both groups, males were predominant (66% Bricker, 70% Wallace). Follow up period was 36.5 months in Bricker group and 17 months in Wallace group. In both groups, the body mass index (BMI) was similar (26.1 kg/m^2^ Bricker and 26.4 kg/m^2^ Wallace). We observed that the stricture rate after performing the Bricker anastomosis technique was 25.3% (19/75) as compared to 7.7% (5/65) after Wallace anastomosis technique, which was statistically significant (*p* = 0.001). In the Bricker group, patients with strictures had higher BMI (28.3 vs. 25.7 kg/m^2^, *p* = 0.05). On average it took 8.5 months in the Bricker group and three months in the Wallace group (*p* = 0.6) to develop stricture.

**Conclusions:**

The stricture rate was significantly higher when Bricker technique was applied. Although the BMI was not different in both groups, patients with a higher BMI were more likely to develop stricture. We believe that the approach of the separate and refluxing technique of Bricker anastomosis especially in obese patients poses a higher risk for anastomotic stricture formation.

## Background

Bladder cancer is the second most common malignancy of urological cancers. In 2017 about 20.000 men and women were diagnosed with bladder cancer in Germany [1]. Approximately only one third of these patients will have muscle invasive disease and subsequent radical cystectomy with urinary diversion [2]. The commonly used types of urinary diversion involve a segment of bowel, typically the ileum, which is then anastomosed to the ureter [3]. However, this ureteroenteric anastomosis is at risk for developing strictures. The postoperative stricture rates range from 3 to 10%; the etiology of which is attributed to local ischemia caused by poor neovascularization after excessive dissection for inflammation, or urinary leakage leading to further scarring of the affected ureter [4]. Thus, a uni- or bilateral obstruction of the ureter may subsequently lead to renal hydronephrosis impairing renal function [5].

The most commonly used forms of ureteroenteric anastomosis techniques are the Bricker (separate) and the Wallace (conjoined) anastomosis. The Bricker anastomosis was first described by Eugene M. Bricker in 1956 whereas David Wallace described his technique in the British Journal of Urology in 1966 [6, 7]. But until today, there have only been four publications and one meta-analysis that evaluate the benefits and harms of each implantation strategy of the ureter [8–12].

The aim of our study was to retrospectively evaluate the outcomes of these two different techniques that were performed on 140 patients without preoperative hydronephrosis between 2009 and 2014. The mean follow up was two years. We sought to identify potential risk factors for developing ureteral stricture and to analyze the incidence and pathologic variables that are associated with ureteroenteric strictures after urinary diversion.

## Methods

Written informed consent to participate in the study was obtained from all participants. The local database was reviewed for patients who underwent radical cystectomy and had either Bricker or Wallace ureterenteric anastomosis performed on them. Only patients without preoperative hydronephrosis or radiotherapy were included.

Each procedure was performed by an experienced surgeon. All cystectomy cases were conducted utilizing open surgical approach. The decision whether to perform Bricker or Wallace anastomosis was dependent upon surgeons’ preference, one using Bricker (T.J.) other using Wallace (S.W.) anastomosis.

All patients underwent heterotopic urinary diversion (ileal conduit). Routinely, patients were discharged from hospital after seven to ten days when leakage from anastomosis could be excluded through radiography. Further follow-up consisted of ultrasound every three months until one to two years after procedure, every six months for another two years, and annual ultrasounds thereafter.

Evidence of hydronephrosis was identified by ultrasound, loopogram, and intravenous pyelogram (IVP); in selected cases by renal scintigraphy depending on patient renal function. A computed tomography was done to exclude extrinsic obstruction. in some cases. If extrinsic obstruction was evidenced, patients were excluded. Only patients with proven narrowing of the ureteroanastomotic region were included in the study. Time until diagnosis and type of treatment was recorded and was defined as time to stricture presentation from the date of surgery e.g. cystectomy.

Statistical analysis was performed using SPSS statistics (IBM SPSS Statistics 25). For comparison of categorical variables Fisher’s exact test and chi-square tests were used. Kaplan-Meier analysis and Log-rank test were used to compare potential treatment success or failure by risk factors on univariate analysis. Univariate Cox proportional hazard regression models were performed for association of significant variables to identify independent predictors of stricture formation.

## Results

Our study examined 140 patients, of which 75 patients underwent Bricker anastomosis and 65 patients underwent Wallace anastomosis, following radical cystectomies and urinary diversion for urothelial malignancies (urothelial carcinoma. Patients who received either radiation therapy or neoadjuvant chemotherapy were excluded.

The demographics of the two cohorts are depicted in Table 1. The median age for both the Bricker and the Wallace group was 71 (mean age in Bricker group 70 and 69.8 in Wallace). The percentage of males in the Bricker group was 66 and 70% in the Wallace group.

**Table 1:** Comparison of patients undergoing Bricker or Wallace anastomosis

	Bricker	Wallace
	*n* = 75	*n* = 65
Surgeon	J.H.	S.W.
Male	50 (67%)	46 (70%)
Female	25 (33%)	19 (30%)
Median Age	71	71
Smoking history	24 (32%)	21 (32.3%)
Hypertension	47 (62.6%)	41 (63.1%)
Diabetes type 2	5 (6.6%)	4 (6.1%)
BMI kg/m^2^	26.2	26.4
Pathologic stage		
No tumor	5 (6.6%)	4 (6.2%)
Tumor		
≤ T2	46 (65.7%)	40 (65.6%)
≥ T3	24 (34.3%)	21 (34.4%)
Any T N+	9 (12.8%)	7 (11.5%)
Mean preop creatinine mg/dl	1.02	1.01
Mean postop creatinine mg/dl	1.14	1.12
Median est. blood loss/ ml	166	175
Median operating room time/ min	299	285
Median hospital stay/ days	11.3	10.5
Median Follow up months	36.5	17.0

The mean follow-up period was 36.5 months for the Bricker group and 17.0 months for the Wallace group. The body mass index (BMI) was 26.1 kg/m^2^ in patients treated with Bricker and 26.4 kg/m^2^ in patients treated with Wallace anastomosis (*p*-value > 0.05). In the Bricker group, patients who developed stricture had a mean BMI of 28.3 kg/m^2^ and those who did not develop stricture had a mean BMI of 25.7 kg/m^2^ which was statistically significant (*p*-value = 0.05). In the Wallace group, the mean BMI was 26.1 kg/m^2^ in both (stricture and no stricture) groups (Table 2).

**Table 2:** Comparison of patients with or without stricture undergoing Bricker or Wallace anastomosis

Bricker (*n* = 75)			Wallace (*n* = 65)		
	stricture	no stricture	stricture	no stricture	*p*-value
Patients (%)	19 (25.3)^†^	56 (74.7)	5 (7.7)^†^	60 (92.3)	0.001^†^
mean age	69	70	71	72	n.s.
BMI*	28.3*	25.7*	26.1	26.1	0.05*
right side	4	–	1	–	n.s.
left side	6	–	2	–	n.s.
both sides	9	–	2	–	n.s.

The Symbol Renates Tod Thema Bricker Group, means significance was only obtained in the Group with the † or *

The strictures were diagnosed as hydronephrosis in regular postsurgical ultrasound examination after initial Bricker or Wallace implantation. Subsequent radiographic imaging confirmed the presence of an anastomotic stricture.

In the Bricker group 19 out of 75 (25.3%) were diagnosed with hydronephrosis and evidenced to have an ureteroenteric anastomotic stricture (Table 2). In the Wallace group five out of 65 (7.7%) were diagnosed with hydronephrosis and stricture. This was statistically significant (chi-quadrat test, *p* = 0.001) and still seen when Cox Regression after age adjustment was applied (*p* = 0.024). There was no predominant laterality in any of the groups, Bricker or Wallace (Table 2). The median time to develop hydronephrosis was 8.5 months in the Bricker group and three months in the Wallace group (*p* = 0.6).

Of the 19 patients that experienced anastomotic stricture in the Bricker group, 12 received double J stent placement and seven nephrostomy tubes. In the Wallace group one patient had a double J stent and four were treated with nephrostomy tube.

Reporting renal function, as monitored by measuring the serum creatinine level shortly before and two weeks after surgery, the mean creatinine level was 1.02 mg/dl before and 1.14 mg/dl after surgery in the Bricker group. In the Wallace group serum creatinine level was 1.01 mg/dl before and 1.12 mg/dl after surgery. This was not statistically significant (Table 1).

## Discussion

Although Bricker and Wallace anastomotic techniques belong to the most common surgical procedures used in urinary diversion, few studies have addressed their postsurgical outcome [8, 11–14]. Every surgical technique has its advantages and disadvantages, which explains why several techniques have been used to connect the conduit to the distal ureter. However, as only few studies provide sufficient evidence for the superiority of one technique, the decision as to which technique to utilize depends on the surgeons’ own preference.

In our study we have observed that stricture rates were significantly higher in patients receiving Bricker (separate and refluxing) as compared to Wallace (conjoined and refluxing) anastomosis. Kouba et al. have reported on stricture rates that were 2% after Bricker and did not appear after Wallace anastomosis technique in a large series of 186 patients [12]. Follow-up was 34 months and did not differ amongst the two groups. However, in their study, among the group with a higher stricture rate, the BMI was significantly higher (29 vs. 25 kg/m^2^). Obesity and a greater BMI have been proposed as a potential risk factor, because extensive manipulation and mobilization is required, the blood supply is compromised thus unfortunately provoking fibrosis and stricture development. In our study, the mean BMI was almost identical in both groups (26.2 vs. 26.4 kg/m^2^) but a significant difference in the stricture rate was still evident. Further analysis in the Bricker group revealed a higher BMI in the group that developed stricture. In our study, the BMI in the stricture-developing group was 28.3 kg/m^2^ and is thus in accordance with the findings from Kouba et al. Obesity seems to be a risk factor for stricture risk when the Bricker technique is applied.

Other studies, dating back to the 1970s, have not shown one technique being superior to the other. In 1974, Wiederhorn et al. have published a series, comparing 51 Bricker to 15 Wallace type anastomosis [14]. As strictures were not found in the small Wallace group no reliable conclusions could be drawn. In contrast, Esho at al. published their results from 33 Bricker and 33 Wallace anastomosis types also in 1974: After a follow-up of two years, no difference was detected [15]. However, this was a pediatric study and obviously different from the group we are discussing in this study.

A more recent study, published by Evangelidis et al. in 2006, was conducted in a cohort of 237, including post-radiation patients, failed to prove any difference in risk for strictures after Bricker anastomosis. The stricture rate was remarkably low (2.9%) and, although not significant, higher in the Wallace group (4.5%) as compared to the Bricker group (1.8%). Information about mean patient BMI was not provided in this study [8]. In contrast to other studies, no laterality was observed. Several studies have shown that postoperatively, left sided hydronephrosis is predominant and the extensive mobilization of the left ureter required for rectosigmoidal tunneling has been suggested as the etiology [9, 16]. Ischemia at the distal ureter following extensive dissection can lead to fibrosis, scarring and stricture. However, we have not observed this in our study but in the Bricker group 6 had left sided and 4 had a right sided hydronephrosis.

In the view of the rising use of robotic surgery, a study from 2014 published by Desai et al. has addressed various issues when orthotopic neobladder was reconstructed completely intracorporally. Besides being a feasibility study, they reported an overall stricture rate of 3.8% performing Wallace type anastomosis in 65% and Bricker type anastomosis in 35%. It is noteworthy, that even though the follow-up largely differed between 1 month and 9.8 years and operation time was between 6 to 8 h, the resulting stricture rate was similar to the rate observed by using an open approach [13]. A comment in European Urology by Giannarini et al. stated, that this type of surgery should be offered to a well selected group of patients, however a higher risk of stricture formation rate when robotic intracorporal anastomotic technique is applied could be excluded [17].

Of the eligible, published studies, one meta-analysis by Davis et al. has summarized the most relevant issues when Bricker and Wallace techniques are compared [10]. In this analysis, they included a prospective trial conducted by Liu et al., that did not find one technique to be superior to the other [11]. Stricture rate was 3.8% (Bricker) as compared to 2.2% (Wallace) which was not statistically significant in a study group of 99 patients. Interestingly, they also reported a higher stricture rate in patients with elevated BMI (25.2 kg/m^2^) when compared to the group without stricture formation (23.3 kg/m^2^) (*p* = 0.008). In their meta-analysis, Davis et al. concluded that despite complication rates in earlier studies of up to 14%, the Bricker ureteroenteric anastomosis was not associated with a higher rate of ureteroenteric stricture. The authors also stress that follow-up data are only available at short term. However, as ureteroenteric strictures are most likely to develop within the first 12 months following urinary diversion and all publications included in the analysis reported follow up data of between from two to four years. Furthermore, oncologic outcomes did not differ with regard to each of the anastomotic techniques employed, which is consistent with previous published studies. Davis and colleagues concluded that, based upon current available data, the final selection of the diversion method should be based on the surgeons’ experience and preference.

Thus, even today not much can be said about superiority or inferiority of either technique in demonstrating a higher or lower risk for stricture formation. This might relate to the retrospective character of all these studies or heterogeneous patient groups. However, in line with the data published by Liu et al. and Kouba et al., we suggest higher risk for stricture building in patients with elevated BMI [11, 12].

Addressing the question of time to first appearance of hydronephrosis, no conclusion can be drawn due to the small number of patients in our study that became symptomatic in the Wallace group (*n* = 5). One could hypothesize, that the wide spatulation of the ureters will build up more scare tissue and thus has an impact on building of fibrotic tissue which takes longer than the direct attachment of the ureter ends to the bowel segment. If narrowing becomes apparent in the Wallace group, it might be related to early building of scar tissue in the anastomotic region.

Interestingly, neither anastomotic technique affected the patients’ overall renal function. Postoperative creatinine levels four weeks after surgery and removal of the stents remained stable and did not differ significantly from the levels prior surgery.

There are some notable limitations to the study: firstly, the study was conducted in a retrospective manner and the mean follow up of the groups differed. However, as noted above, strictures at the ureteroenteric anastomosis typically present within 12 months after surgery. In this study, the median follow-up of the shorter follow-up group (Wallace) was 17 months. In their study of 124 patients undergoing open ureteroenteric anastomotic revision, Packiam et al. have shown a median time to stricture of 9 months [18]. Secondly, as there were two different surgeons each performing only one type of technique, it is impossible to separate the outcomes from the influence of the surgeon. In the absence of a prospective, randomized trial with a single surgeon, many studies have compared the outcomes of a specific surgical technique (Bricker vs. Wallace) among multiple surgeons. Sha et al., reported retrospectively on 100 patients operated by eight different surgeons and Evangelidis et al. published their study with five surgeons. In both studies, the decision whether to perform Wallace or Bricker technique was dependent upon surgeons’ choice [4, 8].

## Conclusion

This is one of the few studies that have dealt with the incidence rate of anastomotic strictures by comparing two widely used techniques. We conclude, that there is a significantly higher stricture rate when Bricker anastomosis is performed which seems to be even more likely in obese patients. The findings of this study have led to a change: Whereas Bricker technique has been used before, nowadays the less harmful Wallace technique is employed at our institution.

## Data Availability

The data can be received from the corresponding author or the biometrician upon request.

## Abbreviations

BMI: Body mass index
IVP: Intravenous pyelogram
